# Preparation, Characterisation and Antibacterial Activity of Carvacrol Encapsulated in Gellan Gum Hydrogel

**DOI:** 10.3390/polym13234153

**Published:** 2021-11-27

**Authors:** Adila Mohamad Jaafar, Norafida Hasnu, Zulkarnain Zainal, Mas Jaffri Masarudin, Mohd Mokrish Md. Ajat, Min Min Aung, Marwah Rayung

**Affiliations:** 1 Department of Chemistry, Faculty of Science, Universiti Putra Malaysia, Serdang 43400, Malaysia; norafidahasnu@gmail.com (N.H.); zulkar@upm.edu.my (Z.Z.); 2 Unit of Chemistry, Centre of Foundation Studies for Agriculture Science, Universiti Putra Malaysia, Serdang 43400, Malaysia; minmin_aung@upm.edu.my; 3 Department of Cell and Molecular Biology, Faculty of Biotechnology and Biomolecular Science, Universiti Putra Malaysia, Serdang 43400, Malaysia; masjaffri@upm.edu.my; 4 Department of Veterinary Pre Clinical Science, Faculty of Veterinary Medicine, Universiti Putra Malaysia, Serdang 43400, Malaysia; mokhrish@upm.edu.my; 5 Institute of Forestry and Forest Products, Universiti Putra Malaysia, Serdang 43400, Malaysia; marwahrayung@yahoo.com

**Keywords:** encapsulated carvacrol, polymer, gellan gum hydrogel, antibacterial activity, *E. coli*

## Abstract

Recently, the antibacterial properties of Carvacrol (Carv) have been significantly reported. However, due to the unstable properties of Carv under various environment conditions, research approaches tailored towards its widespread and efficient use in various antimicrobial applications are scarce. Here, we discuss progress towards overcoming this challenge by utilising the encapsulation of Carv in gellan gum hydrogels to form thin films (GG-Carv) containing different concentrations of Carv (0.01–0.32 M). FTIR spectrum of GG-Carv revealed that both functional groups from GG and Carv existed. The carbon, hydrogen and nitrogen elemental analysis further supported the encapsulation of Carv with the changes in the element percentage of GG-Carv. Both swelling and degradation percentage increased with time and the decreasing patterns were observed as the concentration of Carv increased. In an antibacterial study, GG-Carv exhibited significant antibacterial activity against *E. coli* with the clear inhibition zone of 200 mm and the detection of bacterial growth showed enhancement with continuous decline throughout the study as compared to free-standing Carv.

## 1. Introduction

The essential oils from plant materials are potentially useful as a reservoir for antimicrobial compounds. They are well known for having wide applications in folk medicine, fragrance industries, preservation and food flavouring. As recognised in recent reports, essential oils containing compounds such as carvacrol, eugenol and thymol (phenolic compounds) demonstrate high antibacterial potential [1]. The antimicrobial properties of the plant volatile oils and their constituents have been explored and they extend use of these plants in potential applications such as medical procedures, cosmetics, food and pharmaceutical industries [2]. The most compelling finding is that essential oils are agents for alternative approaches towards controlling the spread of pathogenic organisms as well as in the mitigation of the development of antibiotic resistance [3].

In this context, we highlight carvacrol (Carv), which is found in the aromatic leaves and flowering plant of both thyme (*Thymus vulgaris*) and oregano (*Origanum vulgare*). Interestingly, Carv has shown effective antibacterial activity and has been proven to be a potential agent in the treatment of infections, safe for human and animal health [4]. The researchers worldwide have investigated the wide spectrum of the antibacterial activity of Carv against various types of microorganisms such as *C. albicans* [5], *P.fluorescens* [6], *L.plantarum, S.cerevisiae, B.cinerea* [7], *S.aureus* [8], *Clostridium perfringens* [9], *Salmonella enterica* [10], *L.monocytogenes* and *E.coli* [11]. This monoterpenic phenol has also emerged for its multiple of biological properties such as anti-microbial [12], effective adjuvant for preterm labour [13], bio-film inhibitor [14], anti-inflammatory [15], antioxidant [16], antitumor [17], analgesic [18], anti-parasitic [19], anti-mutagenic [20], anti-cancer [21], anti-viral [22] insecticidal [23] and fish dietary additives [24].

The host, hydrogel, is a three-dimensional, hydrophilic, polymeric network that is capable of imbibing a large amount of water. In order to maintain the three-dimensional structure, the polymer chains of hydrogels are usually cross-linked either chemically or physically [25]. It is highly permeable to various drug compounds, able to withstand acidic environments and has high swelling properties, which can release entrapped molecules through their web-like surfaces [26]. The component of hydrogel, gellan gum, is a microbial polysaccharide that is derived from *Sphingomonas elodea*, previously known as *Pseudomonas elodea*. Significantly, gellan gum is nontoxic, biocompatible and biodegradable, and the resulting hydrogels are transparent and stable [27]. To date, this biopolymer-based hydrogel has been gaining substantial attention as a potential carrier in controlled release studies [28].

Based on the foregoing, it is believed that the encapsulation technology provides stability and protection to enhance the effectiveness due to the fact that Carv is unstable in harsh environment conditions. Carv is volatile, easily evaporates and prone to degradation during the processing, owing to direct exposure to heat, pressure, light or oxygen [29]. The goal of this study is to characterise Carv encapsulation efficiency in a hydrogel polymer system—gellan gum (GG)—as an antibacterial agent. In essence, the embedding of active hydrophobic compounds (chemical immobilisation or physical encapsulation) requires the use of emulsifying techniques to produce the hydrophilic films via aqueous dispersions to compensate for its lack of solubility. Previous studies by Yadava [30] and Vargas [31] encapsulating lovastatin and thyme essential oil, respectively, report similar processes. The Carv is encapsulated in biodegradable gellan gum hydrogel as an alternative way to extend its shelf life and control the release manner, thereby maximising the usage of the compound. Thus, in this study, the research was carried out to prepare the Carv encapsulated in gellan gum hydrogel in the form of a thin film and further characterise it for antibacterial application.

The effectiveness of Carv against *E. coli* is well established [32]. This antibacterial activity of Carv has been attributed to its considerable effects on the structural and functional properties of cytoplasmic membrane [7]. The mechanism of action is believed to be associated with the damage to the cell membrane. Thus, due to the hydrophobic nature of carvacrol, it interacts with the lipid bilayer of the cytoplasmatic membrane and aligns itself between the fatty acid chains. This causes the expansion and destabilisation of the membrane structure, increasing the fluidity and permeability, which finally inhibit the cell growth [33]. A high degree of swelling resulted in a higher water content. This affects the increase in fluidity and permeability and thereby has high expectation to inhibit cell growth [34].

## 2. Materials and Methods

### 2.1. Materials

The chemicals used in this study were glycerine (1,2,3-Propanetriol) (>96%), gelzan (gellan gum) (>96%), carvacrol (2-Methyl-5-(1-methylethyl)-phenol) (>98%) and calcium chloride (CaCl_2_) (>96%). All chemicals were obtained from Sigma-Aldrich (St. Louis, MO, USA). Sodium dihydrogen orthophosphate (NaH_2_PO_4_) (≥98%) was purchased from BDH Chemicals Ltd. Poole, UK, sodium hydrogen carbonate (NaHCO_3_) (≥99.8%) was provided by Fisher Brand (Leicestershire, UK), sodium chloride (NaCl) (≥99%) was obtained from AnalaR (Poole, UK) and potassium chloride (KCl) (≥99.5%) was purchased from HmbG Chemicals (Darmstadt, Germany). All chemicals were used directly without any purification. The strain of *Escherichia coli* (*E. coli* Dh5alpha), the plasmid of which carries gene resistance against ampicillin, was used in the antibacterial activity study.

### 2.2. Preparation of Carvacrol Gellan Gum Thin Films (GG-Carv)

GG-Carv was synthesised via in situ drug loading, in which the Carv was first put to disperse in deionised water (18 MΩ cm) to a specific concentration and mixed with the dissolved 1 g of gellan gum. This mixture was then mixed with CaCl_2_ to establish the physical crosslinking. The solution was stirred at 500 rpm using a hotplate set at a temperature of 80 °C and mixed for 2 h to ensure homogeneity, and then 5 mL of glycerine was added as plasticiser. The resulting solution was poured into the petri dish and left in the oven for 48 h at 35 °C and then stored in a desiccator at room temperature for further characterisation.

In this study, GG-Carv was encapsulated with Carv at various concentrations; 0.01, 0.02, 0.04, 0.08, 0.16 and 0.32 M and are hereafter referred as GG-Carv 01, GG-Carv 02, GG-Carv 04, GG-Carv 08, GG-Carv 16 and GG-Carv 32, respectively.

### 2.3. Characterisations

Fourier transform infrared (FTIR) spectra of the samples were recorded in the range of 4000–400 cm^−1^ on a Perkin-Elmer 1752X Spectrophotometer (Llantrisant, UK) with the KBr disc method, in conjunction with the elemental analysis, LECO CHNS-932 Analyser (St. Joseph, MI, USA). The surface morphology and the cross-section of sample were observed by using Variable Pressure Scanning Electron Microscopy (VPSEM). This was used for the examination of the samples that are not compatible with high vacuum conditions. The samples were prepared and freeze-dried at −80 °C for 2 days to expel the moisture. Then, the samples were fixed on the aluminium stubs and gold coated before imaging. The model used in this study was LEO 1455 VPSEM (VPSEM, Kensington, UK).

### 2.4. The Study of Swelling Percentage

In this study, Pseudo Extra Cellular Fluid (PECF) buffer solution was used and for the preparation of the buffer, an amount of 0.68 g of NaCl, 0.22 g of KCl, 2.5 g of NaHCO_3_ and 0.35 g of NaH_2_PO_4_ were dissolved in 100 mL of deionised water. The solution was stirred until fully dissolved and the resulting solution was then adjusted to pH 5.5 using 10% nitric acid.

Water uptake of GG-Carv with the dimensions of 2 cm × 2 cm was measured by weighing the dried films (*W_d_*) prior to immersion in 20 mL of PECF buffer solution with pH 5.5 at room temperature. The subsequent weight was recorded every 24 h. The films were removed after 72 h, wiped gently with a tissue to expel surface solution and then weighed (*W_w_*). The percentage of water uptake was then determined from the equilibrium swelling ratio:

(1) $$Swelling\;Percentage\;\left(\%\right)=\frac{W_{w}-W_{d}}{W_{d}}\times 100$$

where *W_w_* is the weight of wet samples and *W_d_* is the weight of dry samples.

### 2.5. The Study of Degradation Percentage

Degradation of GG-Carv was measured by weighing its initial weight of 1.0 g (*W_i_*) and leaving it on a Petri dish at room temperature. The degradation test was performed at room temperature considering to the potential of dermal applications and the common storage temperature. The subsequent weight was recorded every day until a constant weight (*W_f_*) pattern was observed. The percentage of degradation was then determined from the equilibrium degradation ratio:

(2) $$Degradation\;Percentage\;\left(\%\right)=\frac{W_{f}-W_{i}}{W_{i}}\times 100$$

where *W_f_* is the final weight of samples and *W_i_* is the initial weight of dry samples.

### 2.6. The Study of Antibacterial Activity

The antibacterial efficiency of Carv was evaluated using two methods, which are disc diffusion test and detection of bacterial growth by optical density.

#### 2.6.1. Disc Diffusion Test

This study was evaluated by following the disc diffusion test method. The GG-Carv with a diameter of 6 mm (similar to the disc size) was placed onto the surface of the Luria Bertani (LB) agar plate incorporated with ampicillin (Amp) and seeded with 100 µL of *E. coli* culture. The plates were incubated at 37 °C for 18 h and the noticeable bacterial inhibition zone around the polymeric thin film was then observed and measured. The standard disc with impregnated antibiotics, ampicillin and kanamycin (Kana), were also assayed as the negative and positive control, respectively.

#### 2.6.2. Detection of Bacterial Growth by Optical Density (OD)

##### The Antibacterial Effect on Cell Viability

Here, 10 mL of LB broth incorporated with ampicillin was seeded with 100 µL of *E. coli* and incubated for 16 h prior to the addition of GG-Carv 08 with dimensions of 10 × 10 mm and 0.08 M Carv. The *E. coli* culture without any further addition was used as the negative control. The OD of each tube was then measured for every subsequent hour at a wavelength of 600 nm against the standard medium.

##### The Antibacterial Effect on Cell Growth

Here, 10 mL of LB broth with the addition of ampicillin was seeded with 100 µL of *E. coli* culture. The GG-Carv 08 and 0.08 M Carv was directly placed into the bacterial culture. The OD of each tube was then measured every subsequent hour. The *E. coli* culture without any further addition was used as the negative control.

## 3. Results

### 3.1. Fourier Transform Infrared Spectroscopy (FTIR) Analysis

Shifting or disappearances of the frequency of functional characteristic peaks indicate the interaction between polymer and drug [35]. The hydrophilicity, which was reported to be the main factor in a hydrogel’s ability to swell, is influenced by the presence of hydroxyl, carboxyl, sulphonic, amidic and primary amidic functional groups [36]. Thus, the IR peaks of pure GG and GG with Carv are compared.

**Figure 1:**
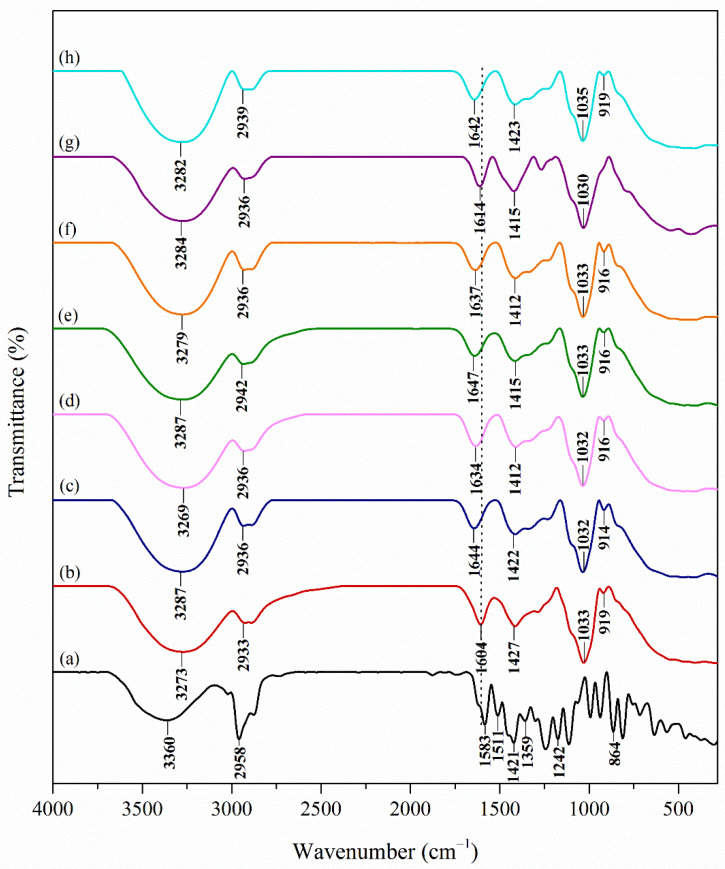
FTIR spectra of (**a**) carvacrol, (**b**) pure GG, (**c**) GG-Carv 01, (**d**) GG-Carv 02, (**e**) GG-Carv 04, (**f**) GG-Carv 08, (**g**) GG-Carv 16 and (**h**) GG-Carv 32.

Chemical structures of the samples were characterised by FTIR (Figure 1). In general, Carv (Figure 1a) showed characteristic peaks at 3360.88 cm^−1^ (phenolic–OH group), 2958.46 cm^−1^ (C–H stretching), 1583 and 1511 cm^−1^ (C–C ring stretching), 1421 cm^−1^ (O–H bending), 1359 cm^−1^ (isopropyl group), 1242 cm^−1^ (C–O stretching) and 864 cm^−1^ (aromatic ring). Meanwhile, the peaks of pure GG (Figure 1b) can be seen at 3273 cm^−1^ (O–H stretching), 2933 cm^−1^ (C–H stretching), 1625 cm^−1^ (C=C stretching), 1427 cm^−1^ (C–H bending), 1033 cm^−1^ (C–O stretching) and 919 cm^−1^ (C–H bending).

From the results obtained (Figure 1c–g), all GG-Carv samples showed peaks in the ranges of 3266–3290 cm^−1^ (O–H stretching) and 2887–2933 cm^−1^ (C–H stretching), which belong to both gellan gum hydrogel and Carv. Furthermore, the peaks at 1637–1642 cm^−1^ and 1408–1415 cm^−1^ (C–C ring stretching), 1033–1036 cm^−1^ (C–O stretching) and 850–918 (aromatic ring), which belong to Carv exist in all GG-Carv samples, reflect the existence of Carv in the gellan gum hydrogel polymer.

### 3.2. Elemental Analysis

Hypothetically, gellan gum contains the carbon and hydrogen; therefore, to determine the carbon content of gellan gum containing Carv, the resulting carbon percentage will be subtracted from the carbon content of free-standing gellan gum. Table 1 shows the weight percentage of carbon, C and hydrogen, H for pure GG and encapsulated GG with various concentrations of Carv. From Table 1, it can be observed that in GG-Carv, the content of C showed an increasing pattern as the concentration of Carv increased. This inclining amount resulted due to the encapsulation of Carv anion and caused the content of C to increase. Similarly, the H content in GG-Carv exhibited the increasing pattern as the concentration of Carv increased.

### 3.3. Variable Pressure Scanning Electron Microscopy (VPSEM) Analysis

VPSEM micrographs were used to study the surface and cross sectional area of GG-Carv. The observation was made at 1000 times magnification. This powerful technique is widely used to capture the characteristic “network” structure in hydrogels [37], encapsulation potential [38], supporting information for release study [39] and crosslinking density [40].

#### 3.3.1. Surface Morphology

**Figure 2:**
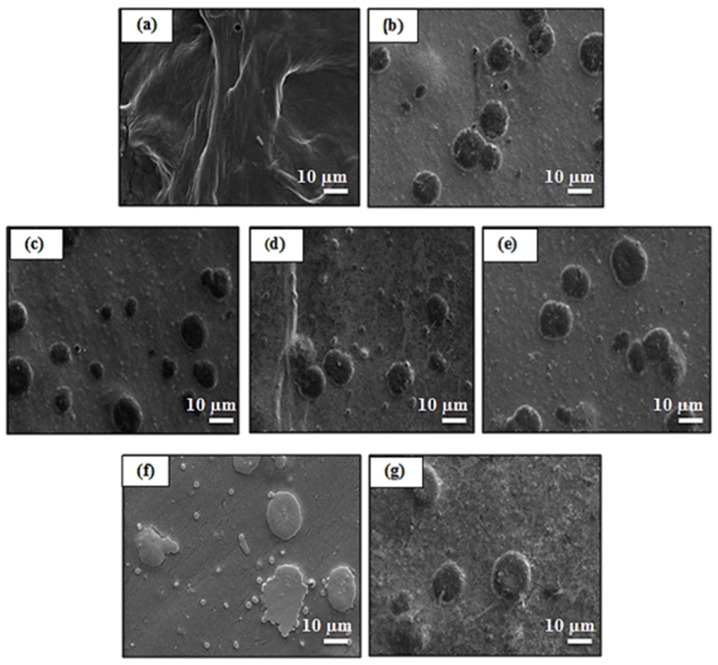
VPSEM surface micrograph at 1000× magnification of (**a**) pure GG film, (**b**) GG-Carv 01, (**c**) GG-Carv 02, (**d**) GG-Carv 04, (**e**) GG-Carv 08, (**f**) GG-Carv 16 and (**g**) GG-Carv 32.

A clear network structure can be observed on the surface morphology of pure GG (Figure 2a). Meanwhile, GG-Carv (Figure 2b–g) exhibited a round-shaped structure scattered evenly, which is possibly due to the Carv binding to the surface of gellan gum hydrogel. The appearances of these structures were more abundant as the concentration of Carv increased with an average diameter of 5 to10 µm.

#### 3.3.2. Cross Sectional Morphology

**Figure 3:**
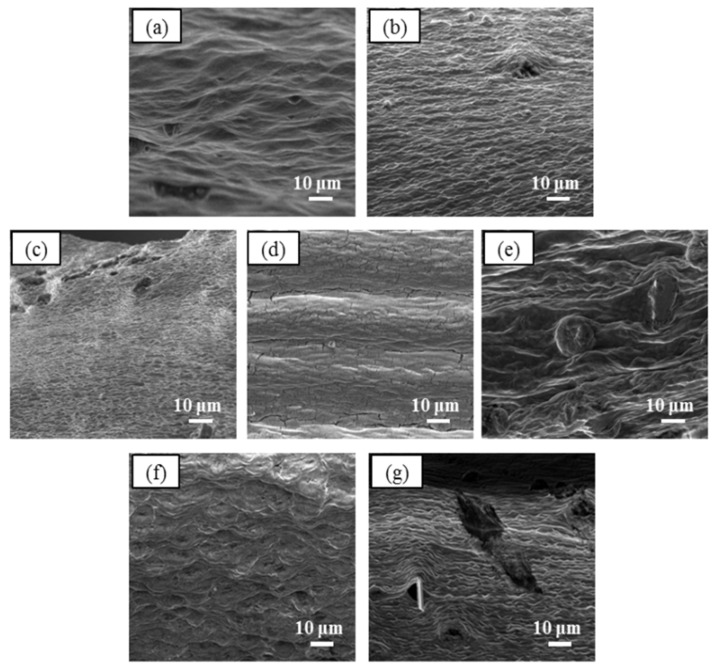
VPSEM cross sectional micrograph at 1000× magnification of (**a**) pure GG film, (**b**) GG-Carv 01, (**c**) GG-Carv 02, (**d**) GG-Carv 04, (**e**) GG-Carv 08, (**f**) GG-Carv 16 and (**g**) GG-Carv 32.

Unpacked layers’ structure can be observed in the cross-sectional morphology of pure GG film (Figure 3a). Meanwhile, GG-Carv (Figure 3b–g) displayed very compact layers as the concentration of Carv increased. This can be explained due to congestion of Carv molecules residing in between the layers.

### 3.4. Swelling Percentage

Hydrogels are three-dimensional, hydrophilic, polymeric networks capable of imbibing large amounts of water or biological fluids [41]. They also provide the effect of becoming a semi-permeable membrane. When hydrogels are treated in an open system and placed in excess solutions, swelling may occur [42]. Similarly, gellan gum has the tendency to incur high swelling behaviours, but this trend will reduce as more Carv is added into the system for encapsulation. In order to accommodate more of the compound, the swelling rate of gellan gum reduces.

**Figure 4:**
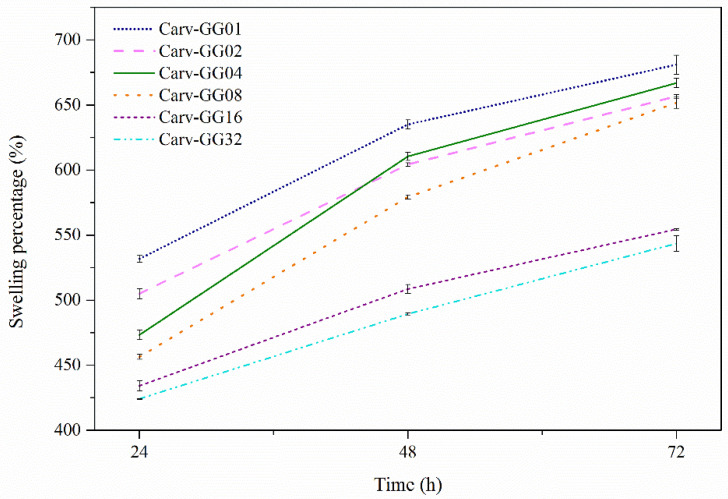
The swelling percentages of GG-Carv 01, GG-Carv 02, GG-Carv 04, GG-Carv 08, GG-Carv 16 and GG-Carv 32.

The result showed that the swelling percentage (Figure 4) increased with time. Table 2 shows the swelling percentage of gellan gum with Carv loading concentrations. As higher concentrations of Carv were used, the absorption of the solutions was less observed. This was reflected in GG-Carv 32, where the highest concentration of Carv caused the lowest swelling percentage due to the formation of more rigid structure of gellan gum hydrogel. Carv is known as a hydrophobic phenolic compound [43]. Thus, the resistance effect towards the solutions which account for the hydrophobicity of Carv also resulted in decreased swelling. Consequently, the higher the concentration of Carv, the higher the expected water resistance of the film.

### 3.5. Degradation Percentage

Most of the degradation study of gellan gum was usually achieved in vivo through the action of enzymes and in vitro [44] in accordance with their application in tissue engineering. However, to understand the degradation behaviour of polymers aimed to be used on the skin, it is important to predict and ultimately be tuned in to their condition at common room temperature for humans. One study reported that a modified gellan gum reacted with methacrylic anhydride and calcium added has an optimised thiol/ene ratio and mixed-mode crosslinking mechanism, which yielded to stiffer hydrogels related to an increase in fibroblast proliferation [45]. Modification of the fabrication of the gellan gum material will truly affect the degradation and the swelling properties in finding the appropriate application.

**Figure 5:**
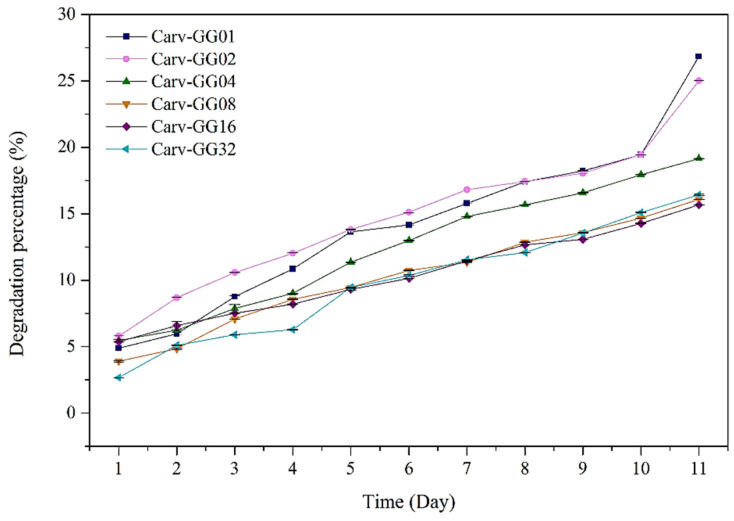
The time-based degradation of GG-Carv at different Carv loadings.

The percentage of degradation (Figure 5) was found to increase with time. However, it was inversely proportional to the concentration. As the concentration of Carv increased, the degradation percentage decreased. Similar to the swelling results, this might be explained by the formation of a more rigid structure of gellan gum hydrogel occurring in GG-Carv at higher concentrations. Hence, this stability resulted in more durable GG-Carv against the environmental conditions as the concentration increased.

### 3.6. The Antibacterial Activity

We chose to evaluate the antibacterial activities of GG-Carv 08 through qualitative and quantitative studies against the same type of bacteria, *Escherichia coli* (*E. coli* Dh5alpha) strain, the plasmid of which carries gene resistance against ampicillin. A qualitative study was conducted by following the disc diffusion method. Meanwhile, the quantitative study was examined through the detection of bacterial growth by optical density (OD) in two conditions, the antibacterial effect against cell viability and cell growth.

#### 3.6.1. Disc Diffusion Test

**Figure 6:**
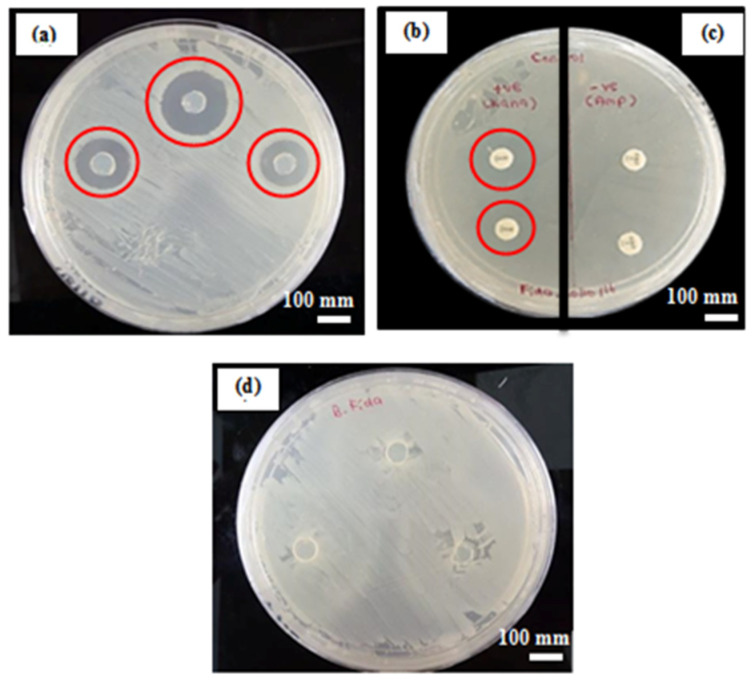
The appearance inhibition zone after 18 h of incubation for (**a**) GG-Carv 08, (**b**) positive control (Kanamycin), (**c**) negative control (Ampicillin) and (**d**) pure GG.

The purpose of the qualitative studies conducted by disc diffusion test was to examine the efficiency of gellan gum hydrogel to release the encapsulated Carv and inhibit bacteria. The inhibitory effects of GG-Carv 08 against *E. coli* were then compared with the standard antibiotics, with kanamycin as a positive control and ampicillin as a negative control. After 18 h of incubation at 37 °C, results showed that pure GG demonstrated no sign of an inhibition zone (Figure 6d), reflecting that GG does not possess antibacterial properties on its own. The same observation was found on the negative control, ampicillin, which also exhibited no sign of an inhibition zone, which proved that the *E. coli* were resistant to ampicillin, and it behaves accordingly (Figure 6c).

In contrast, GG-Carv 08 displayed a significant antibacterial activity with a clear inhibition zone of 200 mm (Figure 6a), which in this case was larger than the inhibition zone of the standard antibiotic, kanamycin, with only 130 mm (Figure 6b). This finding directly highlights the potential of Carv, a natural compound, to be utilised as an antibacterial agent and as an alternative approach to conventional antibiotics. This result also indicates that gellan gum hydrogel works as effective carrier and is capable of releasing carvacrol in a sustained manner within 18 h.

The effectiveness of Carv against *E. coli* is well established [32]. This antibacterial activity of Carv has been attributed to its considerable effects on the structural and functional properties of cytoplasmic membrane [7]. The mechanism of action is believed to be associated with damage to the cell membrane. Thus, due to the hydrophobic nature of carvacrol, it interacts with the lipid bilayer of the cytoplasmatic membrane and aligns itself between the fatty acid chains. This causes the expansion and destabilisation of the membrane structure, increasing the fluidity and permeability, which finally inhibit the cell growth [33]. A high degree of swelling resulted in a higher water content. This affects the increase in fluidity and permeability thereby has a high expectation to inhibit cell growth [34].

#### 3.6.2. Detection of Bacterial Growth by Optical Density (OD)

In order to further investigate the antibacterial activity of GG-Carv 08, the quantitative test was performed through the detection of bacterial growth by optical density (OD) in two conditions, the antibacterial effect against cell viability and cell growth.

##### The Antibacterial Effects on Cell Viability

**Figure 7:**
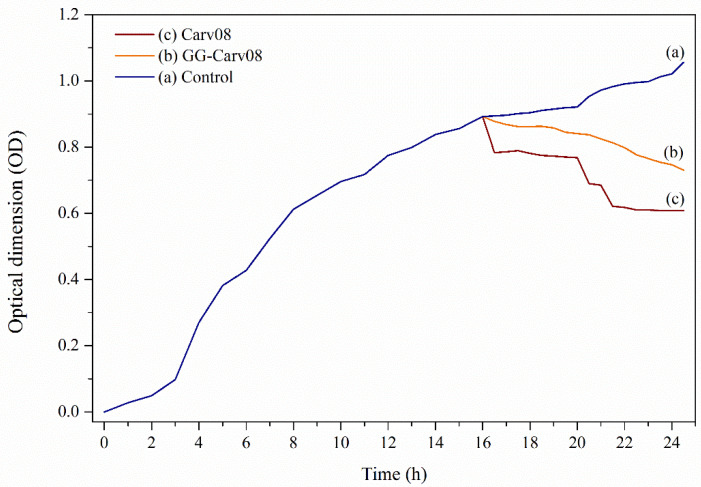
(**a**) The growth profile of *E. coli* culture without further addition and further addition of (**b**) GG-Carv 08 and (**c**) Carv 0.08 M, after 16 h incubation of *E. coli* culture.

This study was performed with the intention to compare the efficiency of the encapsulated GG-Carv with free-standing Carv against bacteria that are in a state of very low metabolic activity and limited available nutrients. Based on the results shown in Figure 6, the growth curve of *E. coli* followed the normal phase of bacterial growth (Figure 7a), which in this case was used as the negative control. Meanwhile, the *E. coli* culture with the addition of free-standing Carv (Figure 7c) exhibited a decrease in bacterial cells but started to remain constant at the 21st hour. This can be explained by the environment conditions that might affect the efficiency of Carv and become deteriorated. As reported previously, Carv is a bioactive compound that easily evaporates and is prone to degradation, owing to its properties when exposed heat, pressure, light or oxygen [29].

Meanwhile, the addition of GG-Carv 08 to the *E. coli* culture (Figure 7b) demonstrated a gradual and continuous decrease in bacteria cells, but in a slow and sustained manner. This trend was expected to continue for extended hours until the maximum release of Carv could be achieved. Above all, this result reflects that the main function of the host, gellan gum hydrogel, had successfully controlled the release of Carv. In further applications, the prolonged antibacterial effect can be expected from GG-Carv to treat infectious bacteria.

##### The Antibacterial Effects on Cell Growth

This study demonstrated the antibacterial effects on cell growth. The growth phase of bacteria is defined as an increase in bacterial number in a population rather than in the size of individual cells. Thus, with the addition of GG-Carv 08 and Carv, the inhibition of the growth and the dead cells are considered to have disrupted or broken membranes due to the antibacterial activity of carvacrol against bacteria. This mechanism of action is believed to be associated with the damage caused to the cell membrane [46]. Thus, this study was performed with the intention to compare the efficiency of the encapsulated GG-Carv with the free-standing Carv against bacteria that are in a state of very high metabolic activity and high nutrients available.

**Figure 8:**
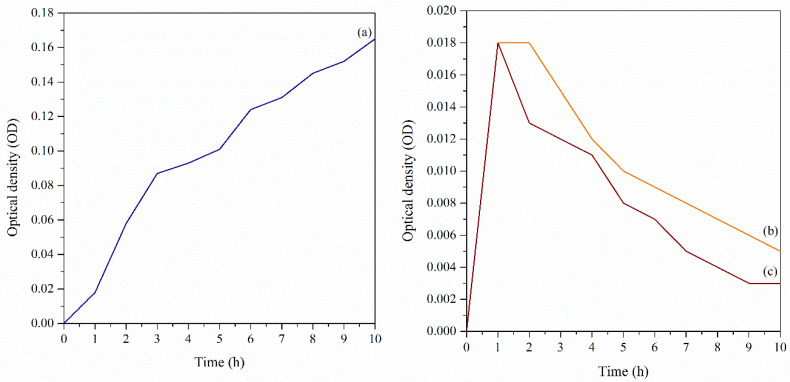
(**a**) The growth profile of *E. coli* culture without further addition and further addition of (**b**) GG-Carv 08 and (**c**) Carv 0.08 M, after 16 h incubation of *E. coli* culture.

According to the results, the growth curve of *E. coli* followed the normal phase of bacterial growth throughout the 10 h (Figure 8a), which in this case were used as the negative control. In contrast, the trends of *E. coli* culture with further addition of GG-Carv and free-standing Carv were similar with previous antibacterial effects on cell viability, respectively, both showing a reduction. The *E. coli* cultures with immersed GG-Carv showed the significant and continued decline of growth but still in controlled manner (Figure 8b). Meanwhile, the *E. coli* cultures with free-standing Carv exhibited a great decline, but started to remain constant at the 9th hour (Figure 8c). The results obtained in this study prove that encapsulated Carv performed better than the free-standing Carv. Hence, it has been proven that the encapsulation technology enhanced the release of Carv and the usage of this compound could be maximised in further applications.

## 4. Conclusions

The preparation of carvacrol encapsulated in gellan gum hydrogel in the form of a thin film (GG-Carv) was successfully achieved. This was confirmed by the FTIR spectrum of GG-Carv, which showed the combination of both functional groups from the hydrogel and Carv. The elemental analysis further supported the encapsulation with the observed changes to the element percentage. Similarly, the swelling percentage and percentage of degradation increased with the time in normal environmental conditions, but showed a decreasing trend with concentrations of Carv being loaded into the system. GG-Carv showed significant antibacterial activity against *E. coli*, with a clear inhibition zone of 200 mm. Subsequently, the detection of bacterial growth through optical density showed enhancement with continuous decline throughout the study as compared to free-standing Carv. Incorporating Carv together with gellan promotes the bioavailability and bio-delivery. Hence, the gellan gum hydrogel is proven to be an effective carrier of carvacrol for antibacterial applications. This study has generated fundamental knowledge of gellan gum hydrogel Carv films which could be used and further explored in appropriate bacterial conditions with enhanced properties for the development of antibacterial applications. Subsequently, this would pave the way towards potential applications as wound-healing patches as well as in the development of more sterile food packaging systems.

## Figures and Tables

**Table 1:** Elemental analysis of weight percentage of carbon, C and hydrogen, H for pure GG and encapsulated GG-Carv with various concentrations of Carv.

Material	Weight Percentage (%)	
	C	H
Pure GG	20.33	8.97
GG-Carv 01	22.52	9.08
GG-Carv 02	23.76	9.29
GG-Carv 04	26.77	9.52
GG-Carv 08	27.42	9.90
GG-Carv 16	28.45	10.21
GG-Carv 32	29.62	10.40

**Table 2:** The effect of Carv loading on swelling percentage of GG.

Materials	Carv (M)	Swelling Percentage (%)
GG-Carv 01	0.01	680
GG-Carv 02	0.02	670
GG-Carv 04	0.04	650
GG-Carv 08	0.08	645
GG-Carv 16	0.16	550
GG-Carv 32	0.32	540

## Data Availability

All data are available in the main text.
